# Alcohol Use as a Function of Physical Activity and Golfing Motives in a National Sample of United States Golfers

**DOI:** 10.3390/nu13061856

**Published:** 2021-05-29

**Authors:** Jimikaye Courtney, Eric Handley, Sherry Pagoto, Michael Russell, David E. Conroy

**Affiliations:** 1 College of Health and Human Development, Pennsylvania State University, University Park, PA 16802, USA; esh12@psu.edu (E.H.); mar60@psu.edu (M.R.); conroy@psu.edu (D.E.C.); 2 Institute for Collaboration in Health, Interventions, and Policy, University of Connecticut, Storrs, CT 06269, USA; sherry.pagoto@uconn.edu

**Keywords:** alcohol drinking, sports, golf, motivation, cancer prevention, social hierarchy

## Abstract

Alcohol and physical inactivity are risk factors for a variety of cancer types. However, alcohol use often co-occurs with physical activity (PA), which could mitigate the cancer-prevention benefits of PA. Alcohol is integrated into the culture of one of the most popular physical activities for adults in the United States (U.S.), golf. This study examined how alcohol use was associated with total PA, golf-specific PA, and motives for golfing in a national sample of golfers in the U.S. Adult golfers (*n* = 338; 51% male, 81% White, 46 ± 14.4 years) self-reported alcohol use, golfing behavior and motives, and PA. Most (84%) golfers consumed alcohol, averaging 7.91 servings/week. Golf participation, including days/week, holes/week, and practice hours/week, was not associated with alcohol use. Golfers with stronger social motives were 60% more likely to consume alcohol. Weekly walking (incident risk ratio (IRR) = 7.30), moderate-to-vigorous PA (MVPA; IRR = 5.04), and total PA (IRR = 4.14) were associated with more alcohol servings/week. Golfers’ alcohol use may be higher than the general adult population in the U.S. and contributes 775 extra kilocalories/week, a surplus that may offset PA-related energy expenditure and cancer-protective effects. Alcohol use interventions targeting golfers may facilitate weight loss and reduce cancer risk, especially for golfers motivated by social status.

## 1. Introduction

Alcohol is a Group 1 carcinogen that directly increases the risk of a multitude of cancers, including increasing the risk of breast and colon cancer by 40–60% [1,2]. Alcohol also represents a calorically dense (7 kcals/g), discretionary source of energy that may promote passive over-consumption of calories [3]. It accounts for up to 10% of total energy intake in adults, and heavier alcohol use increases weight gain and obesity risk [3,4,5,6,7]. Overweight and obesity account for 40% of all cancer diagnoses due to the negative effects of obesity on cancer onset, growth, survival, and metastasis [8,9]. The effects of alcohol consumption on obesity indirectly increase cancer risk [8,9]. Given that over half of U.S. adults consume alcohol [10], alcohol consumption may be a potential behavioral target for addressing energy imbalance, obesity, and cancer risk.

While alcohol use is associated with increased risk for obesity and many cancers, physical activity (PA) is associated with lower body mass index and reduced cancer risk [11,12]. Interestingly, despite these opposing effects, research indicates that PA and alcohol use often co-occur, such that physically active adults are more likely to consume alcohol [13,14,15]. This antagonistic clustering of PA with alcohol use is unusual given that health behaviors typically co-occur synergistically just as health risk behaviors tend to co-occur [16,17,18,19]. This antagonistic co-occurrence of PA and alcohol use means that PA may mitigate the obesity and cancer-related risks of alcohol use or that alcohol use may undermine the protective value of PA against obesity and cancer.

The co-occurrence of PA and alcohol use may be more pronounced in certain types of PA, such as golf, that have incorporated alcohol into the sport’s culture and where the proverbial “19th hole” involves a trip to the bar. Golf is the second most popular sport among U.S. adults, with more than 24 million Americans participating annually (more than the population of every state except California and Texas) [20]. Golf could provide a unique context for examining the antagonistic clustering of PA and alcohol use, but little is known about alcohol use in golfers. This study aimed to characterize alcohol use in golfers and investigate associations between golfers’ alcohol consumption and PA, both in general and specific to golf participation, and whether golfers’ alcohol consumption was associated with the reasons people participate in golf, i.e., their golfing motives, such as golfing for fun, health, competition, or social reasons [21,22,23,24].

Cross-sectional research in youth, college students, and the general population consistently supports a positive association between moderate alcohol use and PA [13,14,15]. This association persists across age groups, levels of PA, and levels of drinking. Longitudinal studies examining within-person associations between alcohol use and PA have mixed findings, with findings indicating a positive [25,26], negative [27], or no association [28,29,30]. Most previous research has focused on adolescents or college-aged students [13,15,28,30] (for an exception see Conroy et al. [25]). Additionally, previous studies have focused on total PA volume or intensity-specific duration [13,15]. Only a handful of studies have tested for differences in alcohol use as a function of sport type, and none has examined associations between alcohol use and the magnitude of participation in a specific type of PA. Given that some types of PA are conducive to alcohol use, examining alcohol use within people who regularly engage in a specific type of PA would shed more light on the PA–alcohol use association.

Golf is potentially compatible with concomitant alcohol consumption given its relatively slow pace and light intensity, and few sports have integrated alcohol as seamlessly into their experience as golf, where golfers can consume alcohol during and after a round. Golf engages a large proportion of the American population, with 24 million Americans golfing at over 14,000 golfing facilities throughout the U.S. [20]. Many golfers cite the social aspect of golfing as a primary reason for their interest and continued engagement in golfing [21,22,23,24]. Golf typically involves small groups, golfers walk or ride in a cart together throughout the course, and many courses include clubhouses that afford opportunities for socializing before or after a round [21,22,23,24]. Some clubs sell alcohol from both the clubhouse and beverage carts that drive around the course. This combination of the social nature of golf and the ready availability of alcohol at courses makes golf an interesting type of PA to study when examining the co-occurrence of PA and alcohol use in adults.

Golfers’ motives for engaging in the sport are broad and include competition with themselves and others, exercise and health benefits, social benefits, relaxation/stress relief, and enjoyment [21,22,23,24]. Likewise, common drinking motives include social, enhancement, coping, and conformity motives [14]. Understanding why individuals engage in golf may deepen researchers’ understanding of associations between PA and alcohol use, particularly if both behaviors share underlying motivational origins [14,27]. The social nature of golf could also influence alcohol use [13]. We propose that motives for participating in physical activities, such as golf, may be linked with alcohol use. Social motives for golf in particular may be associated with greater alcohol use during or after a round of golf. Social behavior can be oriented either toward getting along (i.e., affiliative motives) or getting ahead (i.e., status or dominance motives) [31]. We contend that the strength of these social motives may be associated with alcohol use.

The purpose of this study was to characterize golfers’ alcohol use and identify behavioral and motivational characteristics of golfers associated with higher alcohol use. The three aims involved testing how golfers’ alcohol use is associated with (1) participation in golf-specific activity (e.g., practice frequency), (2) golfing motives, and (3) overall PA volume and intensity-specific durations. We hypothesized that golfers would consume more alcohol if they (1) engaged in more golfing-specific activities, (2) reported higher levels of social motives for golfing, and (3) engaged in more PA overall.

## 2. Materials and Methods

### 2.1. Participants and Procedures

Participants were adult golfers recruited from a Qualtrics survey panel. We purposively sampled across the adult lifespan (using a rectangular age distribution of individuals 18 to 70 years old), by gender (50% male), and by race/ethnicity (75% non-Hispanic White, 25% Hispanic or minority). Individuals who golfed at least 18 holes per month in June–August 2019 were eligible to participate. Participants completed the Qualtrics survey online from 27 May–3 June 2020. This study was approved by the Institutional Review Board of Pennsylvania State University (Protocol #: STUDY00014980). Participants provided informed consent to participate in the study.

### 2.2. Measures

#### 2.2.1. Demographics

Demographic characteristics were assessed using self-reports of age in years, sex (male or female), ethnicity (Non-Hispanic or Hispanic), race (American Indian/Alaska Native, Asian, Native Hawaiian/Pacific Islander, Black, White, or two or more races), marital status (married/cohabitate, widowed, divorced, separated, never married), employment status (never employed, not employed but looking, employed part-time, employed full-time, or retired), and education (no schooling, preschool to Grade 12, high school graduate or equivalent, some college/no degree, Associate’s degree, Bachelor’s degree, Master’s degree, professional degree (Medical Doctor [MD], Doctor of Dental Surgery [DDS], etc.), or doctorate degree). Race was collapsed into a dichotomous variable of White or “Other” race. Marital status was collapsed into a dichotomous variable of married/cohabitate or not married. Employment status was collapsed into three categories: employed full-time, employed part-time, or unemployed/retired. Education status was collapsed into three categories: some college/Associate’s degree, Bachelor’s degree, or post-graduate degree.

#### 2.2.2. Alcohol

Participants reported the total number of servings consumed over the past week by type of alcohol (beer, wine, liquor, non-caffeinated mixed drink, caffeinated mixed drink with regular soda, caffeinated mixed drink with diet soda, and caffeinated mixed drink with an energy drink). Response options ranged from 0 to 15+ servings in increments of 1. Total alcohol servings/week was calculated as the sum of all drink types. Participants were categorized as exceeding the threshold for moderate alcohol consumption if they consumed more than 7 (women) or 14 (men) alcohol servings/week [32].

#### 2.2.3. Golf Participation

Participation in golfing-related activities was assessed separately for spring (March/April/May), summer (June/July/August), and fall (October/November/December). For each season, participants indicated the average number of days/week they played golf (0 to 7 days in increments of one), the average number of holes/week played (0 to 90+ holes in increments of 9), and the average number of hours/week spent practicing golfing at a driving range or putting green (0 to 10+ hours in increments of one). Principal components analysis with oblique rotation revealed three underlying factors for participation in golfing-related activities: (1) days/week, (2) holes/week, and (3) practice hours/week. Based on those results, scores were averaged across seasons to estimate the daily frequency of golfing (*α* = .83), typical holes/week (*α* = .89), and the duration of practice hours/week (*α* = .88).

#### 2.2.4. Golf Motives

Participants rated the importance of 16 different golf motives on a scale ranging from 0 (not at all important) to 5 (extremely important). Sample motives included “playing better than others”, “socializing with my playing partners”, “preventing injury”, “enjoying a leisurely time on the course”, and “challenging myself”. Principal components analysis with oblique rotation reduced responses to four correlated factors: social status (5 items, *α* = .76), health (4 items, *α* = .69), enjoyment (4 items, *α* = .71), and skill (3 items, *α* = .66).

#### 2.2.5. Physical Activity

Past-week PA was measured using the International Physical Activity Questionnaire Short Form (IPAQ-SF) [33]. The IPAQ-SF is a widely used self-reported measure of PA demonstrating acceptable reliability and validity across different populations [34,35]. Participants reported the frequency and average daily duration of past-week vigorous-intensity PA, moderate-intensity PA, and walking. Weekly durations at each intensity level (=frequency × daily duration) were screened for outliers greater than three standard deviations below or above the sample mean. Outliers were all above the sample mean and were winsorized to three standard deviations above the mean for each weekly duration variable (vigorous: *n* = 6, moderate: *n* = 7, walking: *n* = 10). Duration of weekly moderate-to-vigorous PA (MVPA) was calculated as the sum of the winsorized values for weekly vigorous and moderate PA. Participants were classified as meeting PA guidelines if they participated in at least 150 min of moderate PA per week, 75 min of vigorous PA per week, or any combination thereof [36]. In accordance with the IPAQ scoring protocol, total weekly PA volume was calculated as the sum of vigorous, moderate, and walking durations weighted for energy expenditure at each intensity [37]. Due to their non-normal, positively skewed distribution, Box-Cox transformations [38] were applied to normalize the distributions of weekly walking duration (λ = 0.26), weekly MVPA duration (λ = 0.34), and total weekly PA volume (λ = 0.30).

### 2.3. Data Analyses

#### 2.3.1. Quality Assurance

Prior to hypothesis testing, data were screened to identify participants whose data should be excluded from the final analytic data set. In total, 366 individuals completed the Qualtrics survey, and 16 participants were removed during primary screening for providing responses that caused concern regarding the validity of their responses. Participants were removed during primary screening due to providing nonsense responses for write-in variables (e.g., “Kzkzmx”. “Yshysusy”; *n* = 12), selecting the maximum value for weekly servings for all types of alcohol beverages (resulting in an implausible total of 105 drinks per week; *n* = 1), implausible responses to health history questions (*n* = 2), or all of the above (*n* = 1). Primary screening resulted in a sample of 350 participants. Next, as a part of secondary screening, participants who reported playing no golf across all three seasons (i.e., 0 days, 0 holes, 0 practice hours; *n* = 3) were listwise deleted for ineligibility. Secondary screening also examined the primary outcome variable, total alcohol servings/week, for outliers greater than three standard deviations above the mean. Outliers for alcohol servings/week were treated as missing. Due to the fact that alcohol servings/week was the primary outcome variable, any participant with missing data for alcohol servings/week (*n* = 9) was listwise deleted from the data set.

#### 2.3.2. Hypothesis Testing

Regression models were used to examine the associations between demographic characteristics, golf participation, golf participation plus golf motives, weekly walking duration (hours/week), weekly MVPA duration (hours/week), total PA volume (MET hours/week), and alcohol servings/week. In the first step of the analysis, we estimated a series of intercept-only models of alcohol servings/week to determine the best fitting model. The models tested included Gaussian, Poisson, negative binomial, zero-inflated Poisson (ZIP), and zero-inflated negative binomial (ZINB) [39,40]. Akaike information criterion (AIC) and Bayesian information criterion (BIC) values, the log-likelihood ratio test (LRT, for nested models), and the Vuong test (for non-nested models) were used to determine the model of best fit [40,41,42].

In the second step of the analysis we extended the model by adding age (sample mean centered), sex, race, and ethnicity to the logit and count models as demographic predictors of alcohol use, due to variability in alcohol use by age, sex, race, and ethnicity. In the third step of the analysis, the demographics model was expanded by adding five sets of predictors of interest: golf participation (days/week, holes/week, practice hours/week), golf participation plus motives, weekly walking duration, weekly MVPA duration, and total weekly PA volume. Golfing variables and PA were added to the logit and count models. All models were fit in R version 4.0.0 [43].

## 3. Results

**Figure 1:**
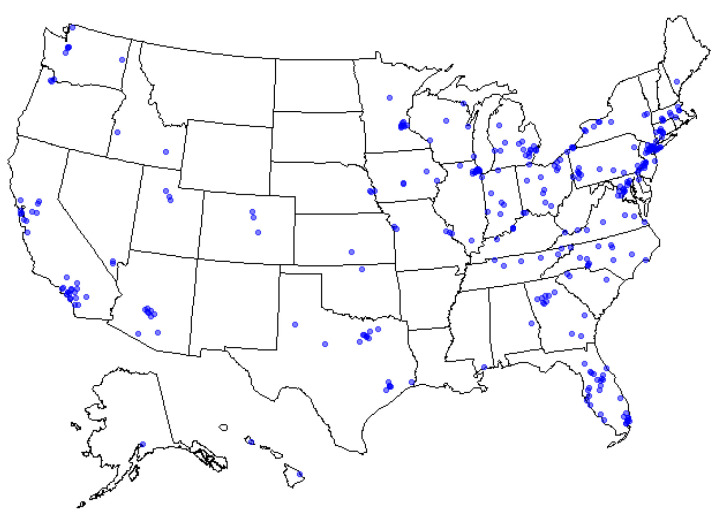
Geographic distribution of sample throughout the U.S.

The final analytic sample comprised 338 participants (51.2% male, 90% non-Hispanic, 81% White) with a mean age of 46 years (standard deviation [SD] = 14.4). Table 1 summarizes the demographic characteristics of the sample. As shown in Figure 1, the sample was recruited from across the United States.

Table 2 summarizes participants’ alcohol servings/week and weekly PA. Most participants (86%) met PA guidelines, and a quarter of all participants exceeded the threshold for moderate alcohol consumption [32].

Table 3 summarizes golf participation and motives. Participants’ golf participation was similar across the three seasons (days/week: *r* = 0.59–0.64, *p* < .01; holes/week: *r* = 0.67–0.79, *p* < .01; practice hours/week: *r* = 0.70–0.73, *p* < .01).

**Figure 2:**
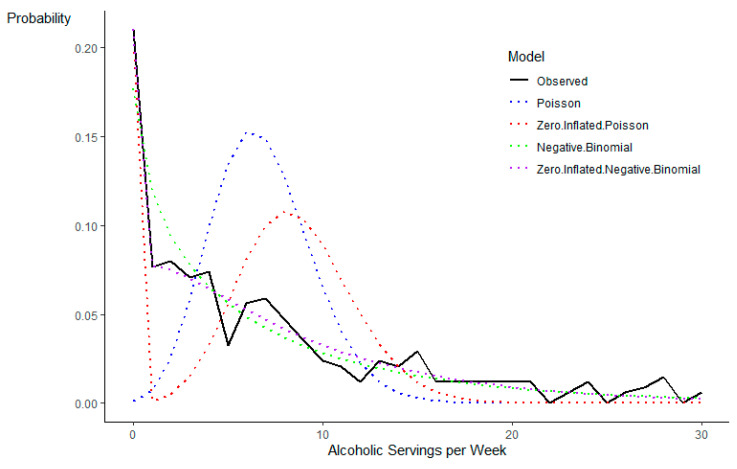
Models for alcohol servings per week.

Fit statistics for the intercept-only model indicated that the ZINB model was the model of best fit. AIC and BIC values were lowest for the ZINB model (AIC = 2004.007, BIC = 2015.476) compared to the Gaussian (AIC = 2307.579, BIC = 2315.225), Poisson (AIC = 3540.811, BIC = 3544.634), negative binomial (AIC = 2014.753, BIC = 2022.399), and ZIP (AIC = 2803.65, BIC = 2811.296). The LRT test confirmed the log-likelihood values were significantly better for the ZINB model than the ZIP model (*Χ*^2^ = 801.64, *p* < .001). The Vuong test confirmed the ZINB model fit better than the negative binomial model (*z* = −1.94, *p* = .03). Figure 2 shows that the ZINB distribution accounted best for the zero-inflation and overdispersion of alcohol servings/week. Therefore, all subsequent analyses treated the outcome variable following a ZINB modeling approach. Post hoc comparisons of model fit confirmed that, when including predictor variables, the ZINB provided the best fit for all models.

In the intercept-only model, participants were estimated to consume 7.91 alcohol servings/week (95% CI: 6.95, 8.91). The baseline odds of participants abstaining from alcohol during the prior week was 0.16 (95% CI: 0.08, 0.24). Table 4 presents the estimated model parameters for the demographics, golf participation, and golf participation plus golf motives. The reference group for each model was participants who were the mean age (46 years), female, non-Hispanic, and White. None of the demographic variables was associated with alcohol servings/week or the odds of abstaining from alcohol during the prior week.

### 3.1. Golf Participation and Motivation

As shown in Table 4, when the three golf participation variables were simultaneously entered into the ZINB model, those variables were not associated with alcohol servings/week or the odds of abstaining from alcohol during the prior week. When golf motives were added to that model, each one unit increase in social status motives increased the odds of a participant consuming alcohol by 60% (95% CI: 0.00, 1.90). None of the other motives for golfing was associated with alcohol servings/week or the odds of abstaining from alcohol during the prior week.

### 3.2. Physical Activity Duration and Volume

Table 5 presents the estimated model parameters for the weekly walking duration (hours/week), weekly MVPA duration (hours/week), and total weekly PA volume (MET hours/week). Each one unit increase in walking duration (Box-Cox transformed) was associated with 7.30 times more alcohol servings/week (95% CI: 1.79, 32.24). Each one-unit increase in MVPA duration (Box-Cox transformed) was associated with 5.04 times more alcohol servings/week (95% CI: 2.38, 12.32). Each one-unit increase in total PA volume (Box-Cox transformed) was associated with 4.14 times more alcohol servings/week (95% CI: 2.17, 7.97). None of the PA variables was associated with odds of abstaining from alcohol during the prior week.

### 3.3. Additional Analyses

Exploratory analyses examined potential moderation of PA associations with alcohol servings per week by demographic variables. In the golf participation model, the negative association between golf days/week and the odds of abstaining from alcohol during the prior week was significantly greater with increasing age (*λ* = 0.10, standard error [SE] = 0.05, *p* = 0.03) (Table S1). In the walking duration model, the effect of walking on increasing servings was significantly smaller in males than females (*β* = −2.61, SE = 1.25, *p* = 0.04) (Table S2). Likewise, total PA volume was positively associated with servings/week, but this association was significantly smaller in males than females (*β* = −1.22, SE = 0.57, *p* = 0.03) (Table S3). Additionally, the positive association between total PA volume and alcohol servings/week was significantly smaller with increasing age (*β* = −0.04, SE = 0.02, *p* < 0.05) (Table S3). Post hoc comparisons of model fit confirmed that, when including predictor variables, the ZINB provided the model of best fit for all models.

## 4. Discussion

The objective of this study was to characterize golfers’ alcohol use and identify behavioral and motivational characteristics of golfers associated with increased alcohol use. Results provided three new insights into the antagonistic co-occurrence of these behaviors.

First, our findings indicate that alcohol use is common among golfers, with over 80% reporting at least one standard serving of alcohol in the past week. By way of comparison, data from the 2007–2016 National Health and Nutrition Examine Survey (NHANES) and the 2018 National Survey on Drug Use and Health indicated that 66% or 55% of adults in the U.S. used alcohol in the past week or year, respectively [10,44]. Although we did not compare our sample with a matched sample of non-golfers, these results suggest that golfers may be more likely to consume alcohol than the general population. Additionally, our sample of golfers were estimated to consume 7.91 alcohol servings/week; whereas, the 2007–2016 NHANES data indicated that adult drinkers consume a median of 1.8 alcohol servings/week. Similarly, Conroy et al. found that adults across the lifespan reported less than 5 alcohol servings per week [25]. As such, it appears that golfers may be more likely to both consume alcohol and to consume a larger volume of alcohol than the general population, making them a potential target for harm reduction interventions [45]. The level of alcohol consumption among golfers, combined with the prolonged sun exposure associated with this activity [46,47], makes golfers an important target for cancer prevention interventions.

This larger volume of alcohol consumption among golfers is also problematic because alcohol is a calorically dense, discretionary source of energy [3]. Assuming that golfers consumed a standard drink with 14 g of alcohol per serving, our sample consumed an estimated 775 kcal/week of discretionary energy from alcohol alone (7 kcal/g alcohol × 14 g alcohol/serving × 7.91 servings = 755 kcal). Previous research suggests that accumulating small energy imbalances over time may increase the risk for obesity, specifically identifying 100 kcal/day as the energy gap in the U.S. that should be targeted for primary obesity prevention [48]. That the golfers in our sample exceeded this 100 kcal/day from alcohol alone is concerning given the potential for alcohol to increase obesity risk in adults after accounting for other risks factors, including PA participation [3,4,5,6,7]. Furthermore, 31% of female golfers and 20% of male golfers were classified as consuming heavy amounts of alcohol (data not shown), which increases weight gain and obesity risk [3,4,5,6,7]. The association of PA with increasing alcohol consumption was also more pronounced in females than males. These findings suggest that PA-related energy expenditure may not offset alcohol-related energy intake even among individuals who regularly engage in a sport, such as golf, that appears to facilitate alcohol consumption. As such, alcohol use in golfers, and particularly female golfers, may undermine the protective value of PA against obesity and cancer risk [8,9].

A second contribution of this study was the finding that social status motives for golf are associated with alcohol use. The social context has been proposed as a potential explanation for associations between alcohol use and PA [13]. Golf is inherently social, and many golfers participate in golf because of its social nature [21,22,23]. We assessed four underlying motives for golfing, including social status, health, enjoyment, and skills. Of these four motives, golfing for social status motives increased the odds of consuming alcohol by 60%. The other three motives were not associated with alcohol use. Golfing for social status represents a more extrinsic form of motivation, and previous work has shown that more extrinsic forms of motivation for alcohol use are more strongly associated with drinking than autonomous motivation [14]. It is also possible that individuals who golf to attain social status may share similar motives for their alcohol use, corresponding with both the proposal that PA and alcohol use may derive from common underlying motives [14], and the finding that individuals who report higher levels of social motives for drinking report drinking more frequently [30]. Finally, alcohol consumption could be a coping mechanism for managing the stress created by seeking greater social status [14,49]. Further research examining golfing and alcohol use motives could help identify the mechanisms underlying the association between golfing for social status and alcohol use.

A third contribution of this research involved the examination of participation in a specific type of PA with alcohol use. Other studies have examined associations between alcohol use and sport participation in general [50,51], but, to our knowledge, these are the first findings to characterize alcohol use among participants in a specific type of PA. The findings provide preliminary support for the assertion that the type of PA an individual engages in may be associated with their alcohol use [13,14]. In the case of golf, alcohol use may be facilitated by the culture of the sport. Along with the ready availability of alcohol at clubhouses and beverage carts, several alcoholic beverages are named after professional golfers, and professional golfers celebrate winning The Open Championship by drinking their favorite alcoholic beverage out of the Claret Jug [52]. Similar to golf, some cycling and running events encourage concurrent PA and alcohol use (e.g., beer mile, hash runs). That alcohol consumption is a fixture of golf culture is concerning given the link between alcohol use and cancer risk [1,2,53,54,55].

The sampling and analytic approaches were noteworthy strengths of this study. This sample was recruited to represent the U.S. population with respect to sex, race, and ethnicity. In contrast, prior work has relied on more homogeneous samples, which were typically limited to adolescents or young adults. The modeling strategy we applied accounted for the distributional features of count-based alcohol use data. This modeling approach, which previous researchers recommended to use when studying MVPA data [56] (which has distributional characteristics similar to alcohol use data), allowed us to address the zero-inflated and over-dispersed nature of alcohol use data. This analytic approach strengthens conclusions by distinguishing the processes that separate teetotalers from drinkers and those that separate light from heavy drinkers. Similar approaches have been applied with intensive longitudinal data but have rarely been applied in cross-sectional analyses of physical activity and alcohol use [25,28,29,30].

Notwithstanding those strengths, individual minority racial groups were not recruited in large enough samples to test whether race moderated associations. The survey also assessed alcohol use over the prior week, and it is unclear how representative that week was of typical consumption (though error variance due to over-/under-reporting alcohol use was assumed to be random). Given that data were collected during the COVID-19 pandemic, participants’ alcohol use may have been higher than normal [57,58], and their PA and golf participation may have been lower than normal [59,60,61,62]. A third limitation was that data were collected via self-report at a single occasion, introducing the potential for recall and self-report biases. Future research should consider incorporating device-based measures of PA, alcohol use, or both. Incorporating intensive longitudinal measures of PA and alcohol use in golfers would also help to uncover the temporality of the associations between PA, golf participation, and alcohol use in golfers, as well as the long-term effects of PA and alcohol use on energy balance, obesity, and cancer risk. It is possible that these findings were due to unmeasured third variables associated with golfing and alcohol use, or they could be an artifact of aggregating behaviors across time. Capturing real-time data would help eliminate these possibilities and identify the temporal sequence of the association between alcohol use and golfing.

## 5. Conclusions

In sum, drawing on a national sample of golfers who were purposively sampled to represent the adult population in sex, race, and ethnicity, this study revealed that alcohol use is frequent among golfers and, consistent with prior research, was greater among those engaged in more overall PA. Although alcohol use was not associated with the intensity of golf engagement, golfers motivated by social status were more likely to consume any alcohol and to consume more alcohol than others. Alcohol use among golfers may represent a public health concern due to its potential to increase discretionary energy intake, promote energy imbalance and weight gain, and mitigate some of the obesity and cancer protective effects of PA. Future research should explore the consequences of alcohol-related energy intake on golfers’ body composition and cancer risk to determine whether golf culture warrants a targeted intervention to decrease obesity and cancer risk.

## Figures and Tables

**Table 1:** Participant demographics.

Demographics	Participants (*n* = 338)
Age in years (Mean ± SD)	46 ± 14.4
Sex (*n* (%))	
Male	173 (51.2)
Female	165 (48.8)
Ethnicity (*n* (%))	
Non-Hispanic	304 (89.9)
Hispanic	34 (10.1)
Race (*n* (%))	
White	275 (81.4)
Other Race ^1^	63 (18.6)
Marital Status (*n* (%))	
Married/Cohabitated	224 (66.3)
Not Married ^2^	114 (33.7)
Employment Status (*n* (%))	
Employed Full-Time	205 (60.6)
Employed Part-Time	35 (10.4)
Unemployed/Retired ^3^	98 (29.0)
Education (*n* (%))	
Some College or less ^4^	131 (38.8)
Bachelor’s Degree	129 (38.2)
Post-Graduate Degree ^5^	78 (23.1)

Notes: SD = standard deviation. ^1^ Other race includes American Indian/Alaska Native (*n* = 4, 1.2%), Asian (*n* = 22, 6.5%), Native Hawaiian/Pacific Islander (*n* = 1, 0.3%), Black (*n* = 30, 8.9%), and two or more races (*n* = 6, 1.8%). ^2^ Not married includes widowed (*n* = 6, 1.8%), divorced (*n* = 31, 9.2%), separated (*n* = 4, 1.2%), and never married (*n* = 73, 21.6%). ^3^ Unemployed/Retired includes “never employed” (*n* = 3, 0.9%), “not employed but looking” (*n* = 25, 7.4%), and “retired” (*n* = 72, 21.3%). ^4^ Some College or less includes “Less than a high school education” (*n* = 1, 0.3%), “High school education or GED” (*n* = 30, 8.9%), “some college/no degree” (*n* = 64, 18.9%), and “Associate’s degree” (*n* = 36, 10.7%). ^5^ Post-Graduate Degree includes “Master’s degree” (*n* = 62, 18.3%), “Professional degree” (e.g., MD, DDS, etc.) (*n* = 9, 2.7%), and “Doctorate degree” (*n* = 7, 2.1%).

**Table 2:** Physical activity and alcohol use behaviors ^1^.

	Range (Min–Max)	Mean ± SD	Median (1QR, 3QR)
Physical Activity Intensity—Hours per Week			
Walking	0–35.5	7.1 ± 8.0	4.6 (2.0, 8.8)
Moderate Physical Activity	0–37.5	6.4 ± 8.0	4.0 (1.5, 8.0)
Vigorous Physical Activity	0–36.2	6.2 ± 7.1	4.0 (1.5, 8.0)
Moderate to Vigorous Physical Activity ^2^	0–64.1	12.8 ± 13.6	8.7 (4.0, 15.6)
Total Physical Activity ^3^	0–104.3	19.7 ± 18.1	14.6 (7.3, 24.0)
Physical Activity Volume per Week ^4^			
Walking	0–117.2	23.5 ± 26.4	15.1 (6.6, 28.9)
Moderate Physical Activity	0–150.2	25.6 ± 32.2	16.0 (6.0, 32.0)
Vigorous Physical Activity	0–290.0	49.4 ± 56.8	32.0 (12.0, 64.0)
Moderate to Vigorous Physical Activity ^5^	0–440.1	75.0 ± 77.2	52.5 (24.0, 96.5)
Total Physical Activity Volume ^6^	0–537.1	98.5 ± 92.4	73.2 (35.7, 125.1)
Alcohol Servings per Week			
Beer	0–15	2.2 ± 3.6	1.0 (0.0, 3.0)
Wine	0–15	1.5 ± 2.3	1.0 (0.0, 2.0)
Liquor	0–14	0.8 ± 1.6	0.0 (0.0, 1.0)
Mixed Drink—All ^7^	0–26	2.3 ± 4.2	0.0 (0.0, 3.0)
Total Servings per Week ^8^	0–31	6.8 ± 7.3	4.0 (1.0, 10.0)

Notes: Min = minimum; Max = maximum; SD = standard deviation; 1QR = first quartile; 3QR = third quartile. ^1^ *n* = 338. ^2^ Sum of winsorized values for vigorous and moderate hours per week. ^3^ Sum of winsorized values for vigorous, moderate, and walking PA hours per week. ^4^ Physical activity volume is based on weighted energy expenditure at each intensity level in MET hours per week [37]. ^5^ Sum of winsorized values for vigorous and moderate MET hours per week. ^6^ Sum of winsorized values for vigorous, moderate, and walking PA MET hours per week. ^7^ Sum total of all four types of mixed drinks: non-caffeinated, caffeinated with regular soda, caffeinated with diet soda, and caffeinated with an energy drink. ^8^ Sum total of all types of alcoholic beverages.

**Table 3:** Golf participation and motives ^1^.

	Overall	Spring	Summer	Fall
Days per Week (Mean ± SD)	1.8 ± 1.2	1.9 ± 1.4	2.0 ± 1.3	1.6 ± 1.4
Holes per Week (*n* (%))				
9 holes/week	-	58 (17.2)	53 (15.7)	57 (16.9)
18 holes/week	-	161 (47.6)	157 (46.4)	133 (39.3)
27 holes/week	-	25 (7.4)	33 (9.8)	26 (7.7)
36 holes/week	-	49 (14.5)	51 (15.1)	40 (11.8)
>36 holes/week	-	23 (6.8)	31 (9.2)	19 (5.6)
Not applicable	-	22 (6.5)	13 (3.8)	63 (18.6)
Holes per Week (Mean ± SD) ^2^	21.1 ± 13.6	21.5 ± 14.6	23.3 ± 15.1	18.4 ± 15.4
Practice Hours/Week (Mean ± SD)	1.8 ± 1.7	1.9 ± 1.9	1.8 ± 1.8	1.6 ± 1.8
Years Golfed (*n* (%))				
Less than one year	12 (3.6)			
1–5 Years	74 (21.9)			
6–10 Years	74 (21.9)			
11–15 Years	31 (9.2)			
More than 15 Years	147 (43.5)			
Golf Motives ^3^				
Social Status (Mean ± SD)	2.4 ± 1.0			
Health (Mean ± SD)	3.6 ± 0.9			
Enjoyment (Mean ± SD)	3.9 ± 0.8			
Skills (Mean ± SD)	3.7 ± 0.9			

Notes: SD = standard deviation. ^1^ *n* = 338. ^2^ Calculated by recoding categorical values for holes per week to numeric values (i.e., 0 = 9 holes/week → 9 holes/week), and then averaging the numeric values. ^3^ Principal components analysis of 16 questions assessing golf motives revealed four underlying factors. Participants’ average scores on each factor were calculated by taking their average for all of the items for a given factor.

**Table 4:** Zero-inflated negative binomial models with demographics, golf participation, and golf participation plus motives predicting alcohol servings per week ^1^.

	Demographics Model	Golf Participation ^2^	Golf Participation + Motives ^2^
Logit Model	λ (SE)	λ (SE)	λ (SE)
Intercept	−1.70 (0.41) **	−0.97 (0.58)	−2.77 (1.99)
Age ^3^	0.03 (0.02)	0.03 (0.02)	0.02 (0.02)
Male	−0.03 (0.44)	−0.09 (0.47)	0.51 (0.61)
Hispanic	−0.44 (1.09)	−0.03 (0.98)	0.22 (0.96)
Other Race	−0.93 (1.01)	−0.89 (1.05)	−14.4 (1027.22)
Golf Days per Week	-	−1.67 (1.70)	−0.79 (0.61)
Golf Holes per Week	-	0.07 (0.08)	0.04 (0.03)
Golf Practice Hours per Week	-	0.09 (0.20)	0.17 (0.22)
Social Status	-	-	−0.52 (0.26) *
Health	-	-	0.81 (0.55)
Enjoyment	-	-	0.17 (0.38)
Skills	-	-	−0.50 (0.42)
Count Model	*β* (SE)	*β* (SE)	*β* (SE)
Intercept	1.88 (0.10) **	1.52 (0.15) **	1.55 (0.31) **
Age ^3^	−0.01 (0.01)	−0.01 (0.01)	−0.001 (0.01)
Male	0.20 (0.12)	0.24 (0.12) *	0.20 (0.13)
Hispanic	0.31 (0.19)	0.27 (0.19)	0.28 (0.19)
Other Race	0.16 (0.15)	0.15 (0.15)	0.11 (0.15)
Golf Days per Week	-	0.01 (0.07)	0.02 (0.07)
Golf Holes per Week	-	0.01 (0.01)	0.01 (0.01)
Golf Practice Hours per Week	-	0.07 (0.05)	0.07 (0.05)
Social Status	-	-	0.04 (0.07)
Health	-	-	−0.05 (0.10)
Enjoyment	-	-	−0.07 (0.09)
Skills	-	-	0.09 (0.09)
Log (theta)	0.30 (0.15) *	0.32 (0.18)	0.30 (0.14) *

Notes: SE = standard error; ** *p* < .01; * *p* < .05. ^1^ *n* = 338. ^2^ Age, sex, ethnicity, and race were included in all models. The reference group is mean age, female, non-Hispanic, and White. ^3^ Age was mean-centered so that a one unit increase in age corresponds with a one-year increase in age above the sample mean (46 years of age).

**Table 5:** Zero-inflated negative binomial models with physical activity intensity and total physical activity volume predicting alcohol servings per week ^1^.

	Walking Hours per Week ^2^	MVPA Hours per Week ^2^	Total PA Volume per Week ^2^
Logit Model	λ (SE)	λ (SE)	λ (SE)
Intercept	−2.43 (0.74) **	−1.14 (0.57) *	−1.45 (1.03)
Age ^3^	0.03 (0.02)	0.02 (0.02)	0.03 (0.02)
Male	−0.01 (0.45)	−0.03 (0.42)	−0.04 (0.42)
Hispanic	−0.06 (1.02)	−0.53 (1.05)	−0.41 (1.18)
Other Race	−9.50 (85.14)	−0.97 (1.00)	−1.64 (2.80)
Walking Hours per Week (Lambda) ^4^	2.99 (2.52)	-	-
MVPA Hours per Week (Lambda) ^5^	-	−1.32 (1.31)	-
Total PA Volume per Week (Lambda) ^6^	-	-	−0.29 (1.37)
Count Model	*β* (SE)	*β* (SE)	*β* (SE)
Intercept	1.43 (0.17) **	1.26 (0.17) **	0.95 (0.24) **
Age ^3^	−0.01 (0.01)	−0.01 (0.01)	−0.01 (0.01)
Male	0.23 (0.12) *	0.20 (0.11)	0.20 (0.11)
Hispanic	0.31 (0.19)	0.26 (0.18)	0.26 (0.19)
Other Race	0.07 (0.15)	0.10 (0.15)	0.06 (0.16)
Walking Hours per Week (Lambda) ^4^	1.99 (0.63) **	-	-
MVPA Hours per Week (Lambda) ^5^	-	1.62 (0.36) **	-
Total PA Volume per Week (Lambda) ^6^	-	-	1.42 (0.33) **
Log (theta)	0.29 (0.13) *	0.44 (0.14) **	0.42 (0.17) *

Notes: SE = standard error; ** *p* < .01; * *p* < .05. ^1^ *n* = 338. ^2^ Age, sex, ethnicity, and race were included in all models. The reference group is mean age, female, non-Hispanic, and White. ^3^ Age was mean-centered so that a one unit increase in age corresponds with a one-year increase in age above the sample mean (46 years of age). ^4^ Walking hours per week was entered into model using the Box-Cox transformed value. ^5^ MVPA (moderate-to-vigorous) hours per week was entered into model using the Box-Cox transformed value. ^6^ Total PA (physical activity) volume per week is based on weighted energy expenditure at each intensity level in MET hours per week [37] and was entered into model using the Box-Cox transformed value.

## Data Availability

The datasets analyzed and/or generated during the current study are available from the corresponding author on reasonable request.

## Supplementary Materials

The following are available online at https://www.mdpi.com/article/10.3390/nu13061856/s1, Table S1: Zero-inflated negative binomial models with golf participation predicting alcohol servings per week and moderation by age, Table S2: Zero-inflated negative binomial models with walking hours per week predicting alcohol servings per week and moderation by age, Table S3: Zero-inflated negative binomial models with total physical activity volume per week predicting alcohol servings per week and moderation by age.
