# Supplementary material for: Alcohol Use as a Function of Physical Activity and Golfing Motives in a National Sample of United States Golfers
Source: Nutrients. 2021 May 29;13(6):1856. doi: 10.3390/nu13061856 (PMC8229716; doi:10.3390/nu13061856)
Supplement: Supplementary file 1 [file nutrients-13-01856-s001.zip › nutrients-1229116-supplementary.pdf]

Table S1. Zero-inflated negative binomial models with golf participation predicting alcohol servings per week and moderation by age [1]

	**Golf Participation** [2]	**Golf Participation*Age** [2]
Logit Model	λ (SE)	λ (SE)
Intercept	-0.97 (0.58)	-0.96 (0.58)
Age [3]	0.03 (0.02)	0.03 (0.04)
Male	-0.09 (0.47)	0.11 (0.48)
Hispanic	-0.03 (0.98)	-0.12 (1.06)
Other Race	-0.89 (1.05)	-0.98 (1.26)
Golf Days per Week	-1.67 (1.70)	-2.16 (0.86)*
Golf Holes per Week	0.07 (0.08)	0.08 (0.04)
Golf Practice Hours per Week	0.09 (0.20)	0.20 (0.23)
Moderation by Age		
Golf Days per Week*Age	-	0.10 (0.05)*
Golf Holes per Week*Age	-	-0.01 (0.00)
Golf Practice Hours per Week*Age	-	-0.02 (0.01)
Count Model	β (SE)	β (SE)
Intercept	1.52 (0.15)**	1.52 (0.14)***
Age [3]	-0.01 (0.01)	0.01 (0.01)
Male	0.24 (0.12)*	0.26 (0.12)*
Hispanic	0.27 (0.19)	0.25 (0.19)
Other Race	0.15 (0.15)	0.16 ().15)
Golf Days per Week	0.01 (0.07)	0.02 (0.07)
Golf Holes per Week	0.01 (0.01)	0.01 (0.06)
Golf Practice Hours per Week	0.07 (0.05)	0.06 (0.05)
Moderation by Age		
Golf Days per Week*Age	-	0.01 (0.01)
Golf Holes per Week*Age	-	-0.00 (0.00)
Golf Practice Hours per Week*Age	-	-0.00 (0.00)
Log (theta)	0.32 (0.18)	0.35 (0.13)**

Notes: SE = standard error; **p<.01; *p<.05. [1] N=338. [2] Age, sex, ethnicity, and race were included in all models. The reference group is mean age, female, non-Hispanic, and White. [3] Age was mean-centered so that a one unit increase in age corresponds with a one-year increase in age above the sample mean (46 years of age).

Table S2. Zero-inflated negative binomial models with walking hours per week predicting alcohol servings per week and moderation by sex [1]

	**Walking Hours per Week [2]**	**Walking hours per Week*Sex [2]**
Logit Model	λ (SE)	λ (SE)
Intercept	-2.43 (0.74)**	-2.73 (1.05)**
Age [3]	0.03 (0.02)	0.03 (0.02)
Male	-0.01 (0.45)	0.43 (1.30)
Hispanic	-0.06 (1.02)	0.03 (0.96)
Other Race	-9.50 (85.14)	-10.92 (215.15)
Walking Hours per Week (Lambda) [4]	2.99 (2.52)	4.19 (3.71)
Moderation by Sex		
Walking Hours per Week*Male	-	-1.78 (5.01)
Count Model	β (SE)	β (SE)
Intercept	1.43 (0.17)**	1.10 (0.23)**
Age [3]	-0.01 (0.01)	-0.01 (0.01)
Male	0.23 (0.12)*	0.80 (0.29)**
Hispanic	0.31 (0.19)	0.32 (0.19)
Other Race	0.07 (0.15)	0.05 (0.15)
Walking Hours per Week (Lambda) [4]	1.99 (0.63)**	3.51 (0.95)***
Moderation by Sex		
Walking Hours per Week*Male	-	-2.61 (1.25)*
Log (theta)	0.29 (0.13)*	0.21 (0.13)*

Notes: SE = standard error; **p<.01; *p<.05. [1] N=338. [2] Age, sex, ethnicity, and race were included in all models. The reference group is mean age, female, non-Hispanic, and White. [3] Age was mean-centered so that a one unit increase in age corresponds with a one-year increase in age above the sample mean (46 years of age). [4] Walking hours per week was entered into model using the Box-Cox transformed value.

Table S3. Zero-inflated negative binomial models with total physical activity volume per week predicting alcohol servings per week with moderation by sex and age [1]

		Total PA Volume per Week – Moderation Models [2]	
	**Total PA Volume per Week [2]**	**Moderation by Sex [2]**	**Moderation by Age [2]**
Logit Model	λ (SE)	λ (SE)	λ (SE)
Intercept	-1.45 (1.03)	-2.60 (1.58)	-2.42 (1.00)*
Age [3]	0.03 (0.02)	0.03 (0.02)	0.12 (0.06)*
Male	-0.04 (0.42)	1.59 (1.70)	-0.08 (0.43)
Hispanic	-0.41 (1.18)	-0.21 (0.98)	-0.47 (1.38)
Other Race	-1.64 (2.80)	-1.51 (1.73)	-12.44 (284.41)
Total PA Volume per Week (Lambda) [4]	-0.29 (1.37)	1.28 (2.01)	1.04 (1.27)
Moderation terms			
Total PA Volume per Week*Male	-	-2.27 (2.30)	-
Total PA Volume per Week*Age	-	-	-0.14 (0.08)
Count Model	β (SE)	β (SE)	β (SE)
Intercept	0.95 (0.24)**	0.46 (0.31)	0.90 (0.21)***
Age [3]	-0.01 (0.01)	-0.01 (0.01)	0.02 (0.01)
Male	0.20 (0.11)	1.02 (0.40)*	0.20 (0.11)
Hispanic	0.26 (0.19)	0.27 (0.18)	0.25 (0.18)
Other Race	0.06 (0.16)	0.07 (0.15)	-0.01 (0.14)
Total PA Volume per Week (Lambda) [4]	1.42 (0.33)**	2.14 (0.45)***	1.49 (0.29)***
Moderation terms			
Total PA Volume per Week*Male	-	-1.22 (0.57)*	-
Total PA Volume per Week*Age	-	-	-0.04 (0.02)*
Log (theta)	0.42 (0.17)*	0.44 (0.15)**	0.40 (0.13)**

Notes: SE = standard error; **p<.01; *p<.05. [1] N=338. [2] Age, sex, ethnicity, and race were included in all models. The reference group is mean age, female, non-Hispanic, and White. [3] Age was mean-centered so that a one unit increase in age corresponds with a one-year increase in age above the sample mean (46 years of age). [4] Total PA (physical activity) volume per week is based on weighted energy expenditure at each intensity level in MET hours per week [37] and was entered into model using the Box-Cox transformed value.
